# Retraction: Clinical and Dermoscopic Evaluation of Periorbital Melanosis and Its Psychological Impact and Effect on Quality of Life: A Descriptive Study

**DOI:** 10.7759/cureus.r223

**Published:** 2026-03-30

**Authors:** Saumya Banchhor, Sanjeev Gupta, Aneet Mahendra

**Affiliations:** 1 Dermatology, Maharishi Markandeshwar Institute of Medical Sciences and Research, Mullana, IND

This article has been retracted by the Editor-in-Chief due to the presence of three irrelevant references, including one reference to a fictitious disease. As a result, the journal's editorial staff no longer has confidence in the accuracy or provenance of the work, thus requiring retraction. 

The authors disagree with the decision to retract.

